# Supplementary material for: Statistical method scDEED for detecting dubious 2D single-cell embeddings and optimizing t-SNE and UMAP hyperparameters
Source: Nat Commun. 2024 Feb 26;15:1753. doi: 10.1038/s41467-024-45891-y (PMC10897166; doi:10.1038/s41467-024-45891-y)
Supplement: Supplementary file 1 — Supplementary Information [file 41467_2024_45891_MOESM1_ESM.pdf]

Supplementary Figures

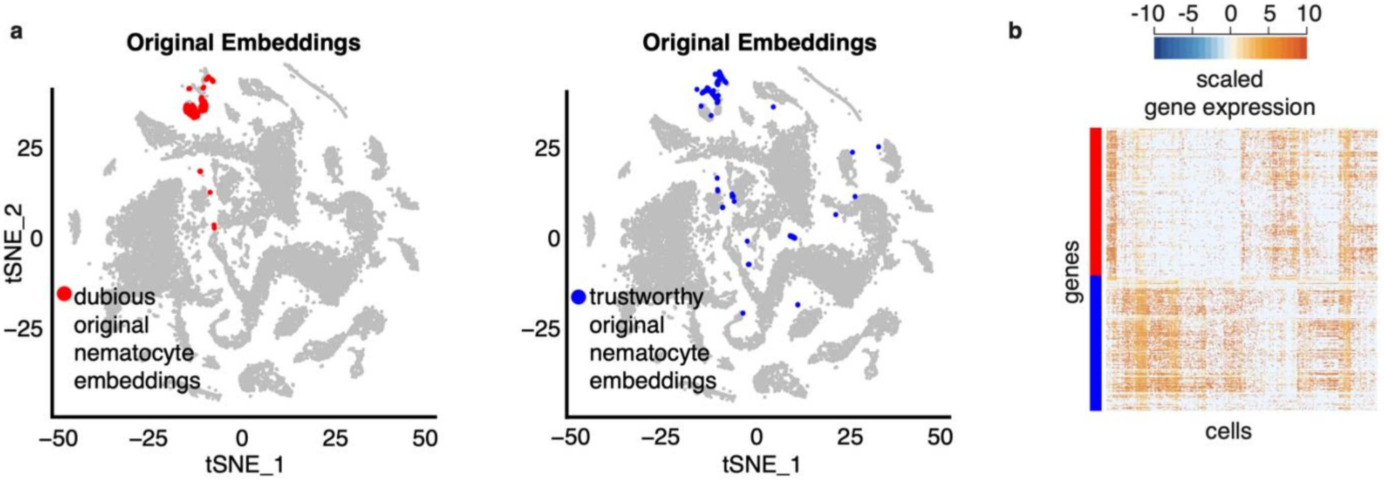

Supplementary Fig. S1. Dubious and trustworthy embeddings of the nematocyte cluster in the Hydra dataset. a, Comparative t-SNE plots at the original perplexity 40 of *nematocyte* cells with dubious (left) or trustworthy cell embeddings (right). **b,** Gene expression heatmap of the highlighted cells in **a.**

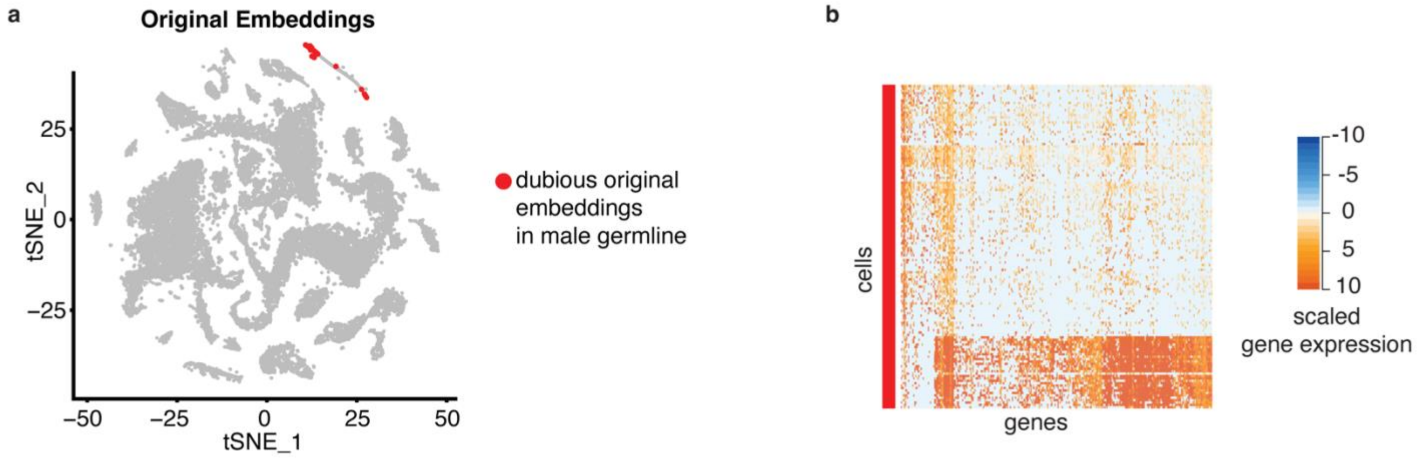

Supplementary Fig. S2. Dubious embeddings of male germline cells in the Hydra dataset. a, t-SNE plot at the original perplexity of 40, with the male germline cells with dubious embeddings highlighted. **b,** Gene expression heatmap of the cells highlighted in **a**.

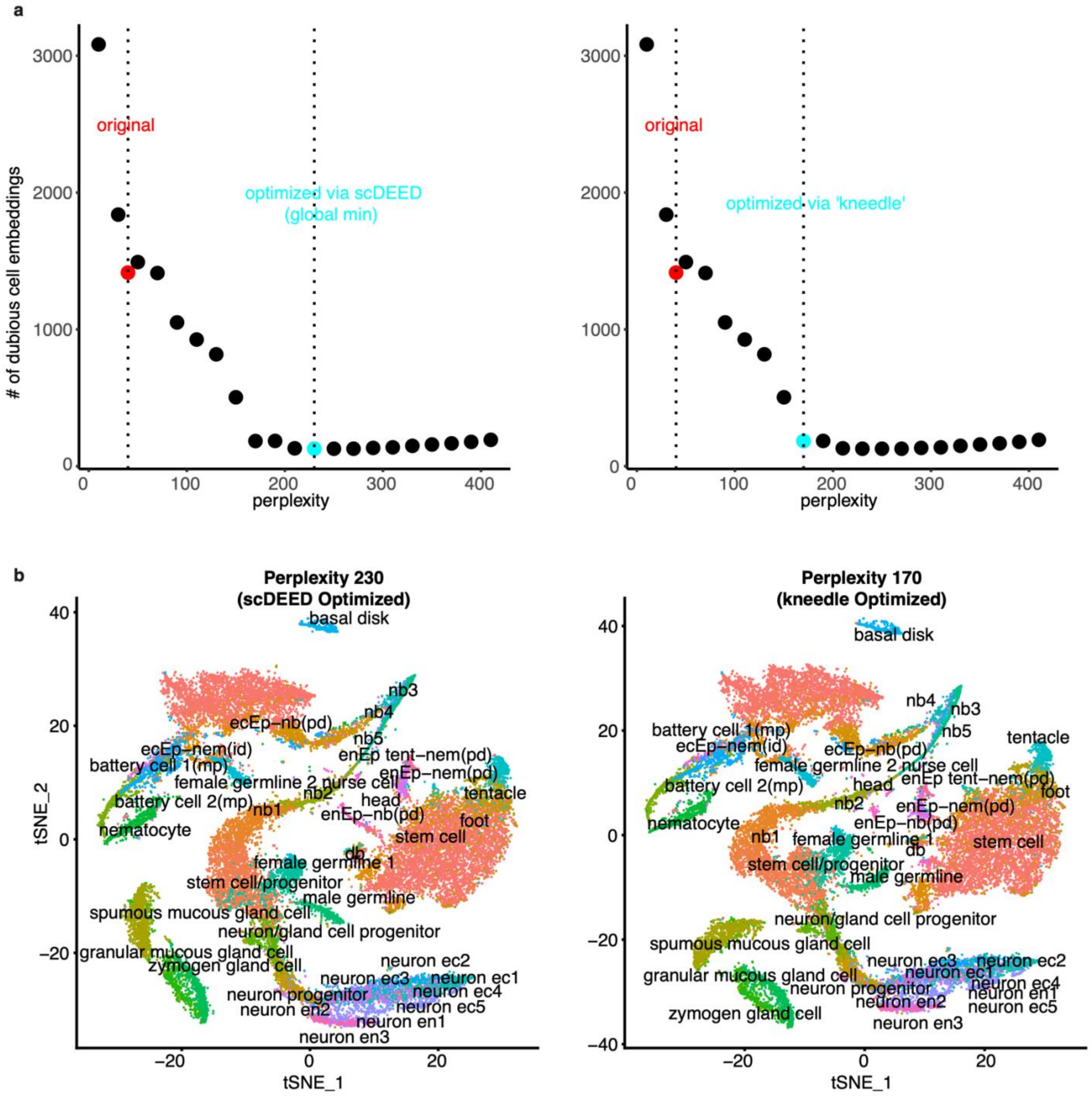

Supplementary Fig. S3. Comparison of optimal perplexity selected by scDEED using two criteria for the Hydra dataset. a, Plots of the number of dubious cell embeddings (the y-axis) versus perplexity (the x-axis) with the original and optimized perplexities highlighted. The optimized perplexity corresponds to the minimum number of dubious embeddings (left) or the elbow point selected by the "kneedle" method [1] (right). **b,** Comparative t-SNE plots corresponding to the optimized perplexities 230 (left) and 170 (right). Source data are provided as a Source Data file uploaded on Zenodo.

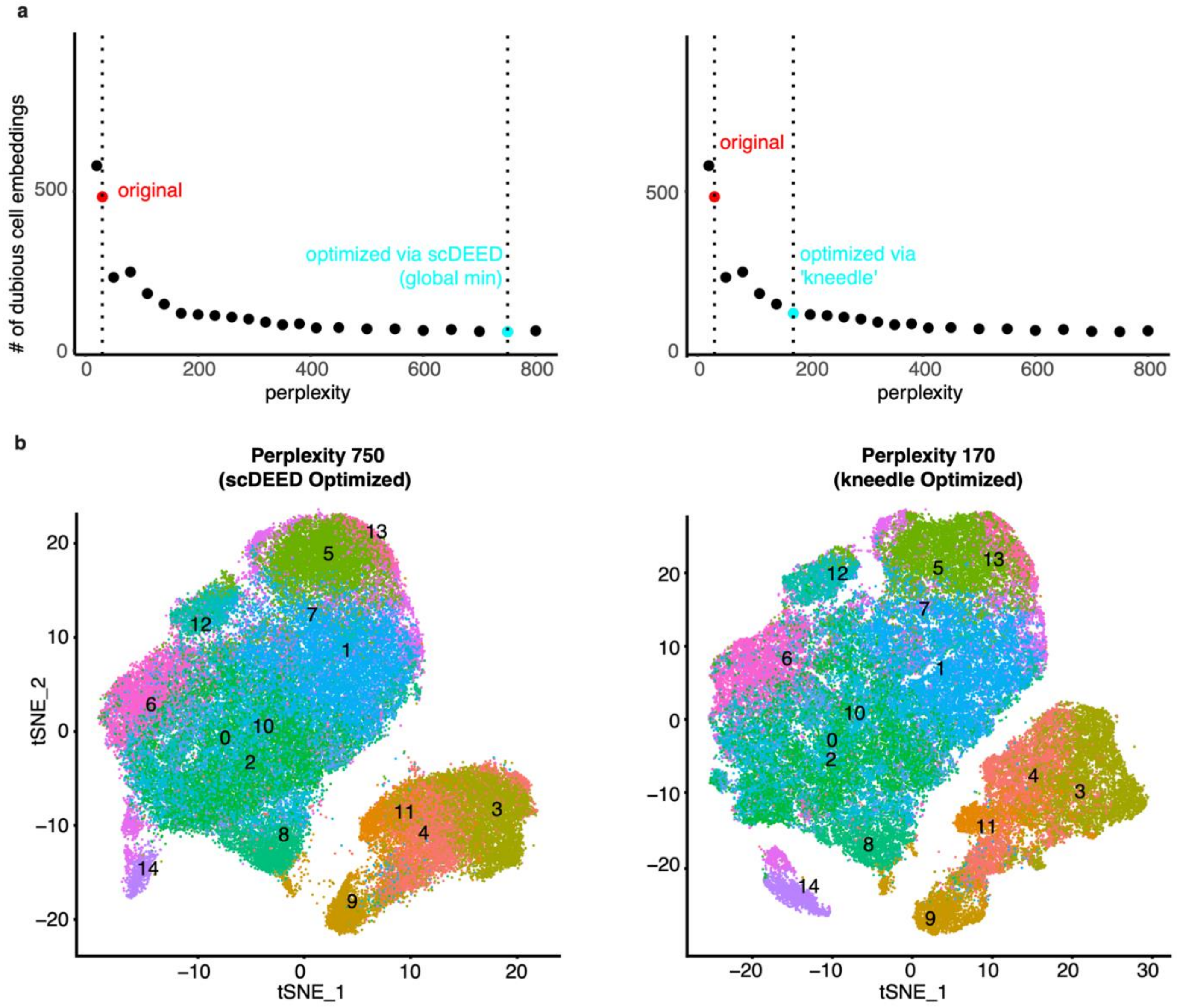

Supplementary Fig. S4. Comparison of optimal perplexity selected by scDEED using two criteria for the CAR-T dataset. a, Plots of the number of dubious cell embeddings (the y-axis) versus perplexity (the x-axis) with the original and optimized perplexities highlighted. The optimized perplexity corresponds to the minimum number of dubious embeddings (left) or the elbow point selected by the "kneedle" method [1] (right). **b,** Comparative t-SNE plots corresponding to the optimized perplexities 750 (left) and 170 (right). Source data are provided as a Source Data file uploaded on Zenodo..

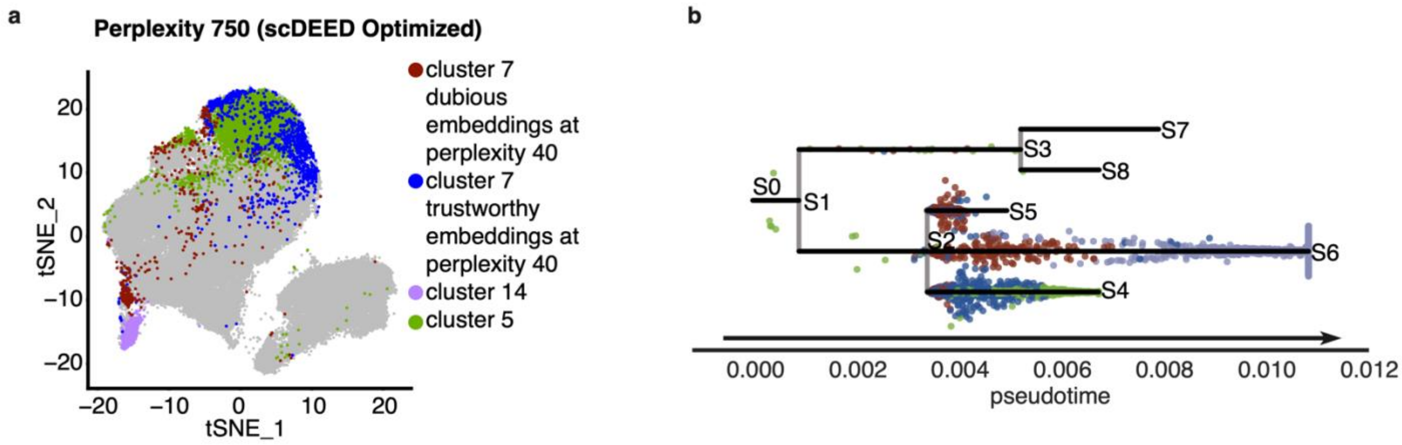

Supplementary Fig. S5. Trajectory analysis of the CAR-T dataset. a, t-SNE plot at the perplexity 750 optimized by scDEED, with *cluster 14* and *cluster 5* highlighted as the reference clusters for *cluster 7*'s dubious and trustworthy embeddings defined by scDEED at the original perplexity 40. **b,** Cell trajectory reconstruction by STREAM, produced by Python functions `st.plot_dimension_reduction()` and `st.plot_branches()` in the Bioconda package "stream" [2]. Source data are provided as a Source Data file uploaded on Zenodo.

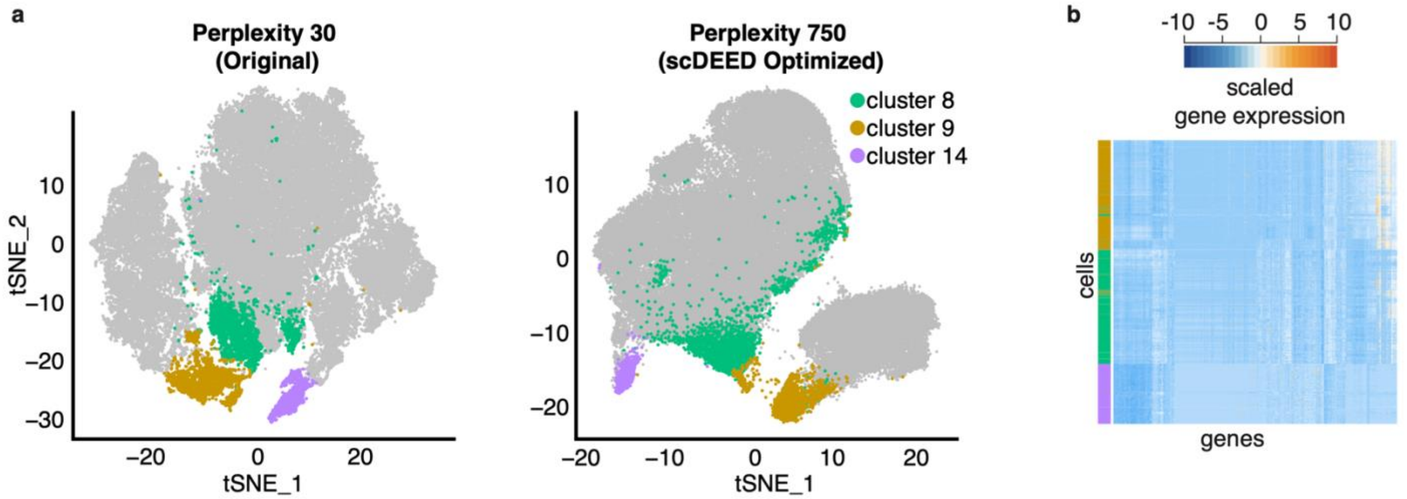

Supplementary Fig. S6. Original and scDEED optimized t-SNE visualizations of *clusters 8, 9* and *14* in the CAR-T dataset. a, Comparative t-SNE plots with *clusters 8*, *9*, and *14* highlighted at the original perplexity 30 (left) and the perplexity 750 optimized by scDEED (right). **b,** Gene expression heatmap of the highlighted cells in **a**, where the cells are ordered by the default hierarchical clustering found by the R function `heatmap.2()`.

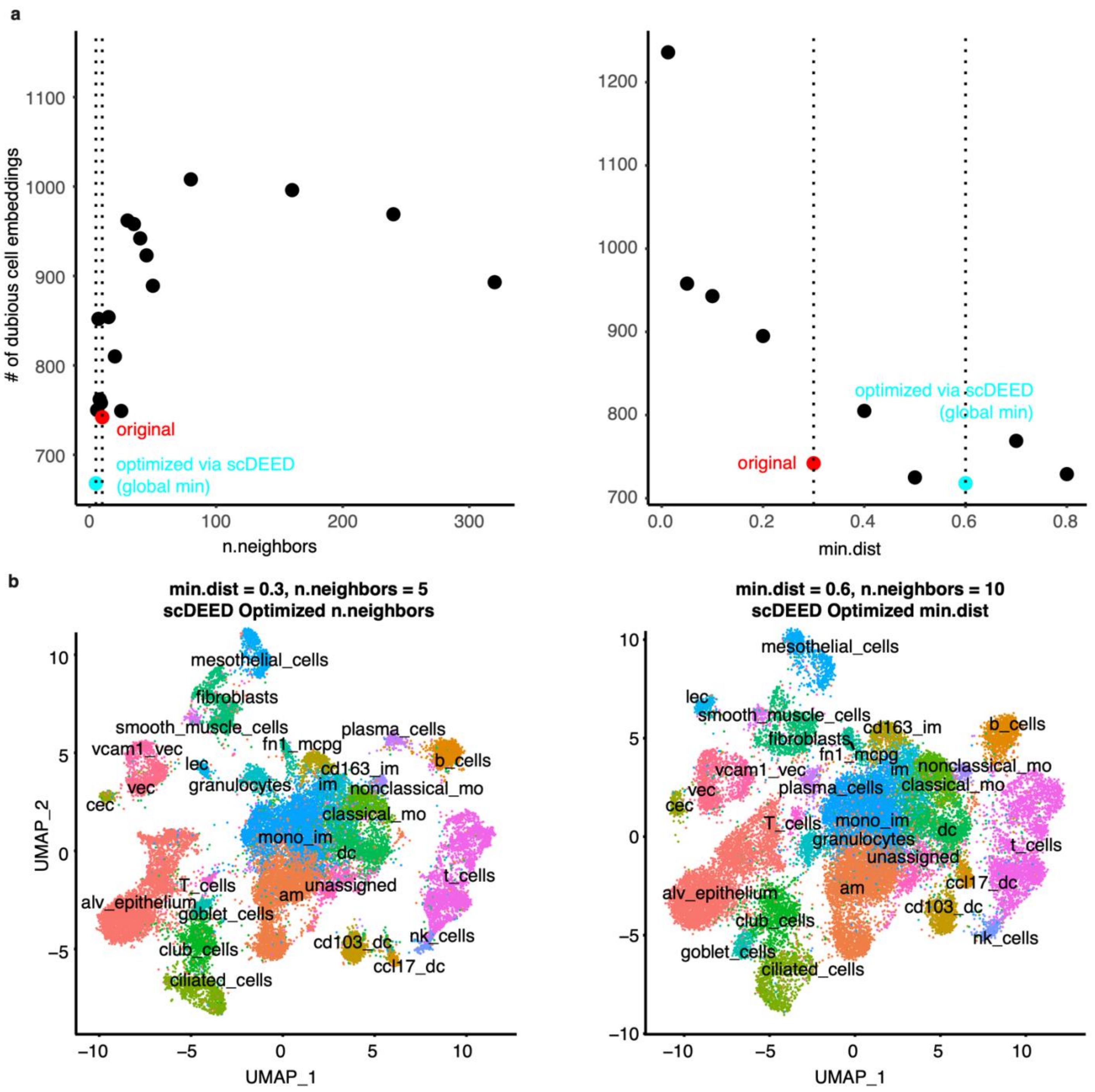

Supplementary Fig. S7. Marginal optimization of UMAP hyperparameters for the Alveolar dataset. a, Plots of the number of dubious cell embeddings (the y-axis) versus n.neighbors (the x-axis) with the fixed min.dist = 0.3 (left) and the number of dubious cell embeddings (the y-axis) versus min.dist (the x-axis) with the fixed n.neighbors = 10 (right), having the original and the optimized hyperparameters highlighted. **b,** Comparative UMAP plots at the marginally optimized n.neighbors by scDEED (min.dist = 0.3 and n.neighbors = 5; left) and the marginally optimized min.dist by scDEED (min.dist = 0.6 and n.neighbors = 10; right). Source data are provided as a Source Data file uploaded on Zenodo.

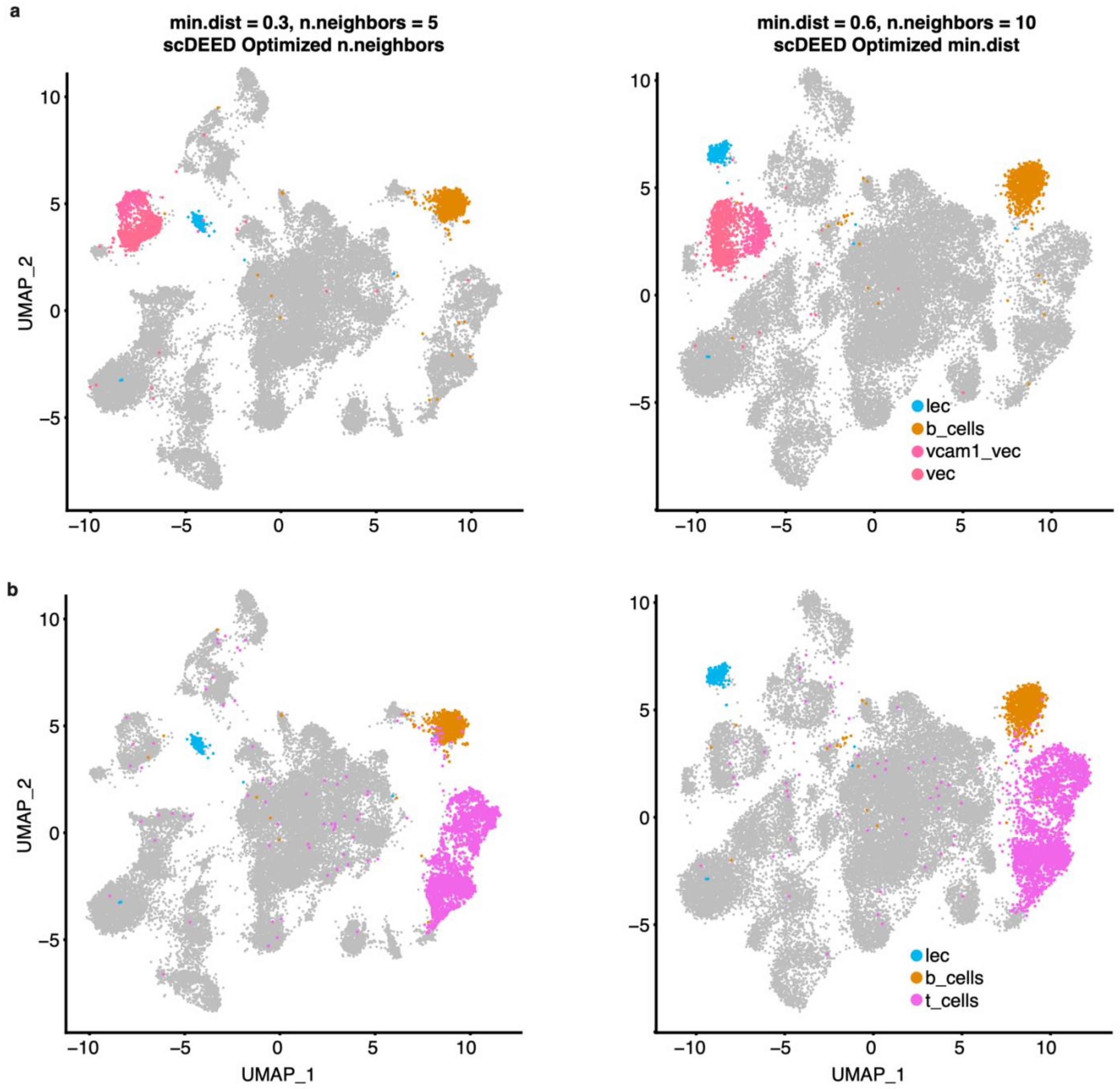

Supplementary Fig. S8. Marginal optimization of UMAP hyperparameters for the Alveolar dataset with highlighted cell types. a, Comparative UMAP plots of the Alveolar dataset with the *lec*, *b_cells*, *vcam1_vec*, and *vec* cells highlighted at the marginally optimized n.neighbors (min.dist = 0.3, n.neighbors = 5; left) and the marginally optimized min.dist (min.dist = 0.6, n.neighbors = 10; right). **b**, Comparative UMAP plots of the Alveolar dataset with the *lec*, *b_cells*, and *t_cells* cells highlighted at the marginally optimized n.neighbors (left) and the marginally optimized min.dist (right).

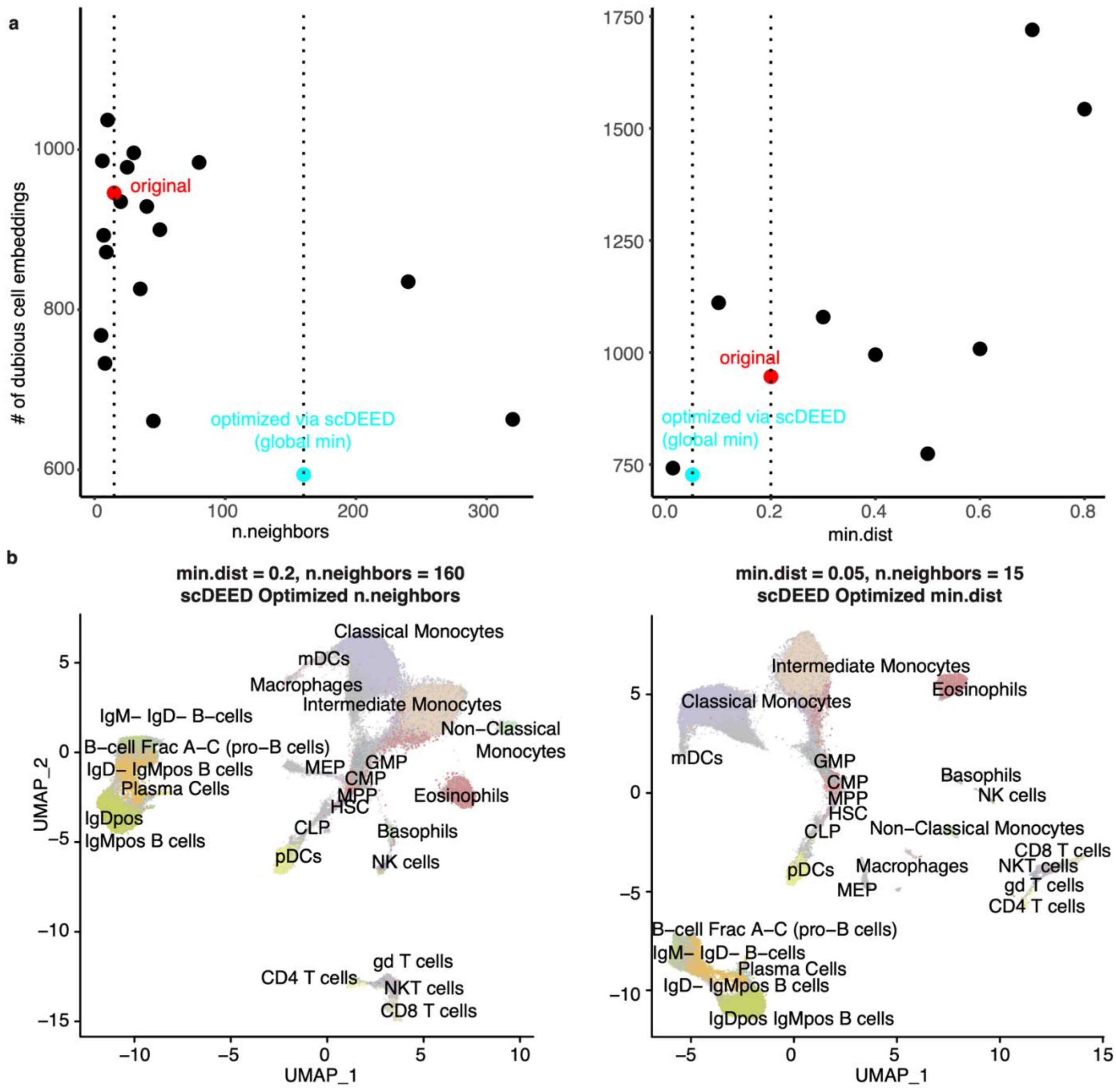

Supplementary Fig. S9. Marginal optimization of UMAP hyperparameters for the Samusik dataset. a, Plots of the number of dubious cell embeddings (the y-axis) versus n.neighbors (the x-axis) with the fixed min.dist = 0.2 (left) and the number of dubious cell embeddings (the y-axis) versus min.dist (the x-axis) with the fixed n.neighbors = 15 (right), having the original and the optimized hyperparameters highlighted. **b,** Comparative UMAP plots corresponding to the marginally optimized n.neighbors by scDEED (min.dist = 0.2 and n.neighbors = 160; left) and the marginally optimized min.dist by scDEED (min.dist = 0.05 and n.neighbors = 15; right). Source data are provided as Source Data files uploaded on Zenodo.

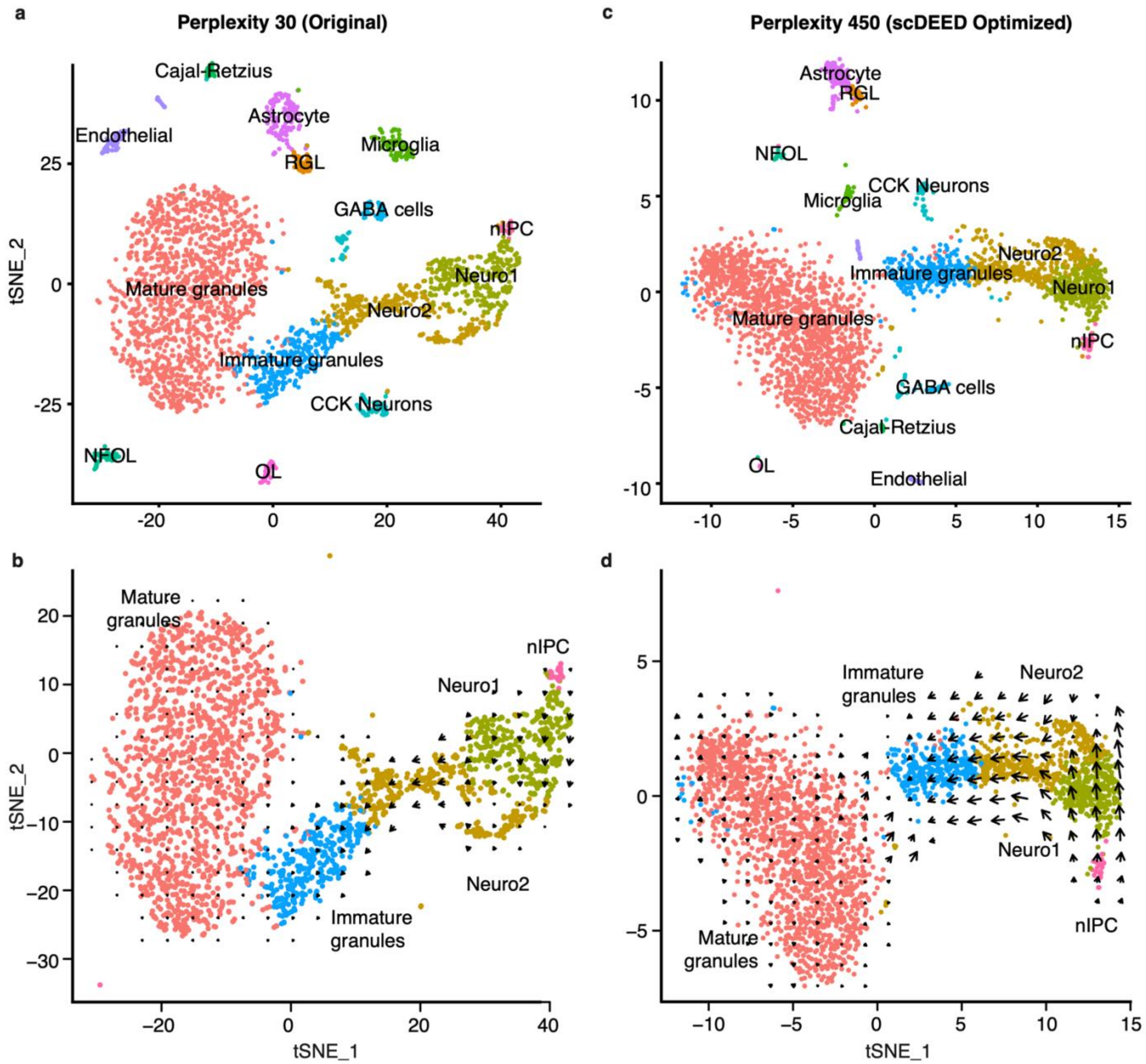

Supplementary Fig. S10. Velocity analysis of the dentate gyrus dataset. a, t-SNE plot at the original perplexity of 30 (OL: oligodendrocytes; NOFL: newly formed oligodendrocytes; RGL: Radial glia-like; Neuro1: Neuroblast1; Neuro2: Neuroblast 2; nIPC: neuronal intermediate progenitor cells). **b,** Velocity analysis using the embeddings at the original perplexity of 30 with the default Velocyto [40] settings. **c** t-SNE plot at the perplexity of 450 optimized by scDEED. **d**, Velocity analysis using the embeddings at the perplexity of 450 optimized by scDEED with the default Velocyto [3] settings.

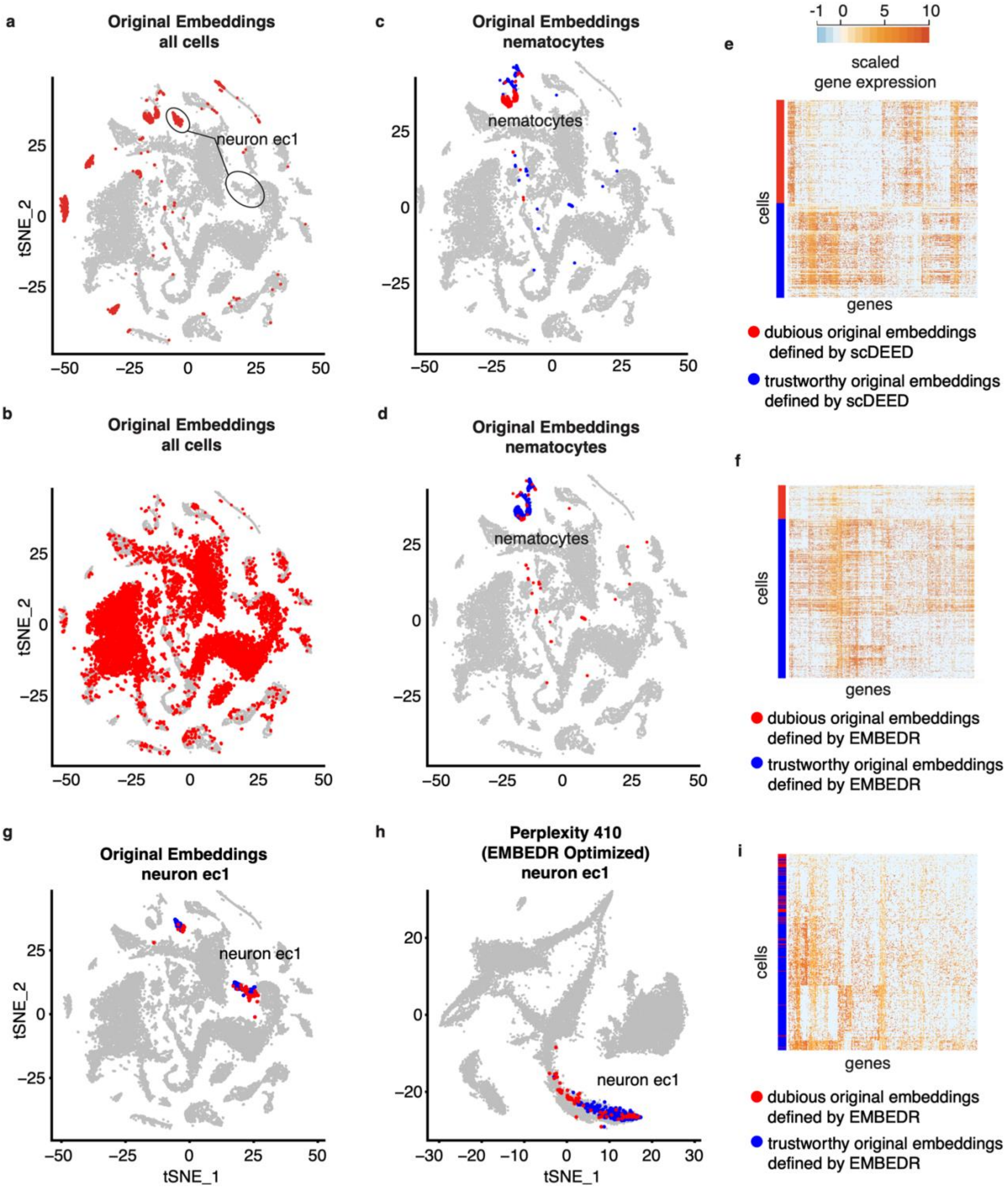

Supplementary Fig. S11. Evaluation of trustworthy and dubious embeddings defined by scDEED or EMBEDR on the Hydra dataset. a–b, Dubious cell embeddings defined at the original perplexity of 40 by scDEED (**a**) or EMBEDR [4] (**b**). **c–d,** Dubious or trustworthy embeddings of *nematocytes* defined by scDEED (**c**) or EMBEDR (**d**). **e–f**, Gene expression heatmaps of *nematocyte*s with trustworthy or dubious embeddings defined by scDEED (**e**) or EMBEDR (**f**). The Euclidean distances calculated in the pre-embedding PC space between the trustworthy- and dubious-embedding cells are greater for scDEED (n = 43,183 cell-cell distances) than EMBEDR (n = 37,824 cell-cell distances) (two-sided t-test, $p < 2.2 \times 10^{-16}$). **g–h,** Dubious or trustworthy cell embeddings of the *neuron ec 1* cluster highlighted in t-SNE visualizations at the original perplexity of 40 (**g**) and the perplexity of 410 optimized by EMBEDR (**h**). **i**, Gene expression heatmaps of the trustworthy or dubious embeddings defined by EMBEDR at the perplexity 40 in the *neuron ec1* cluster.

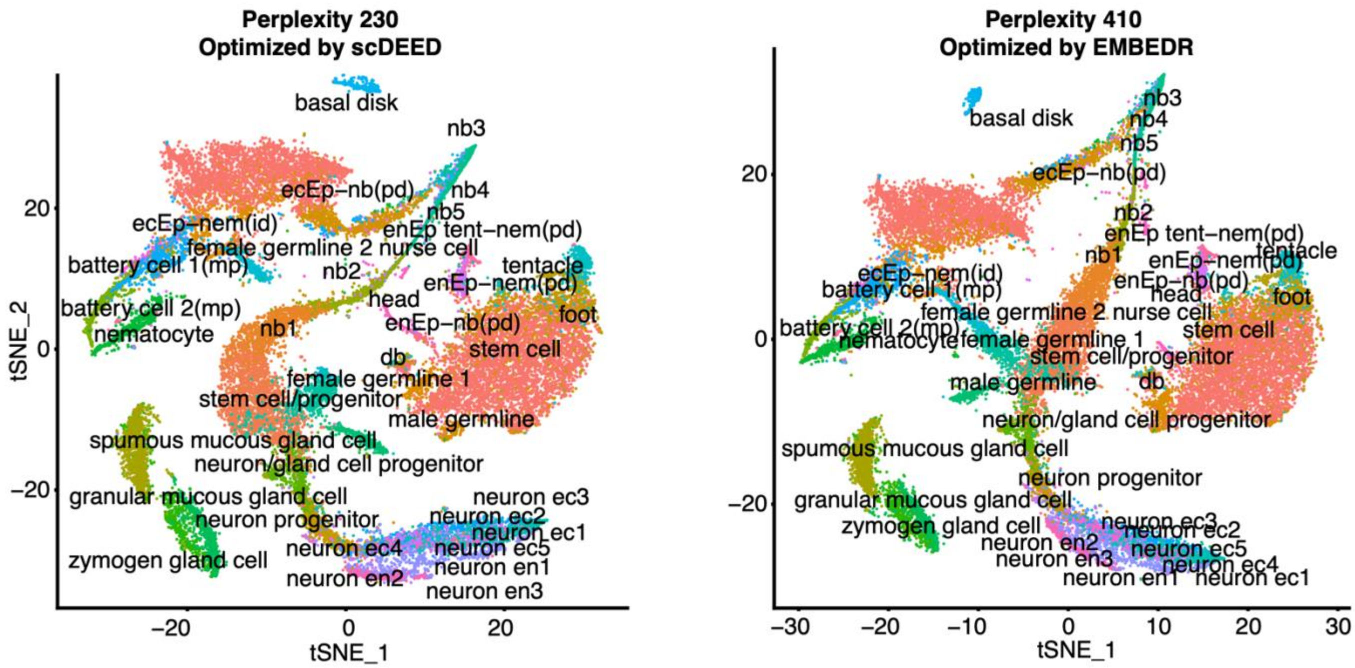

Supplementary Fig. S12. Comparison of t-SNE perplexity optimization by scDEED vs EMBEDR. t-SNE visualizations of the Hydra dataset using the optimized perplexity of 230 found by scDEED (left) and the optimized perplexity of 410 found by EMBEDR (right).

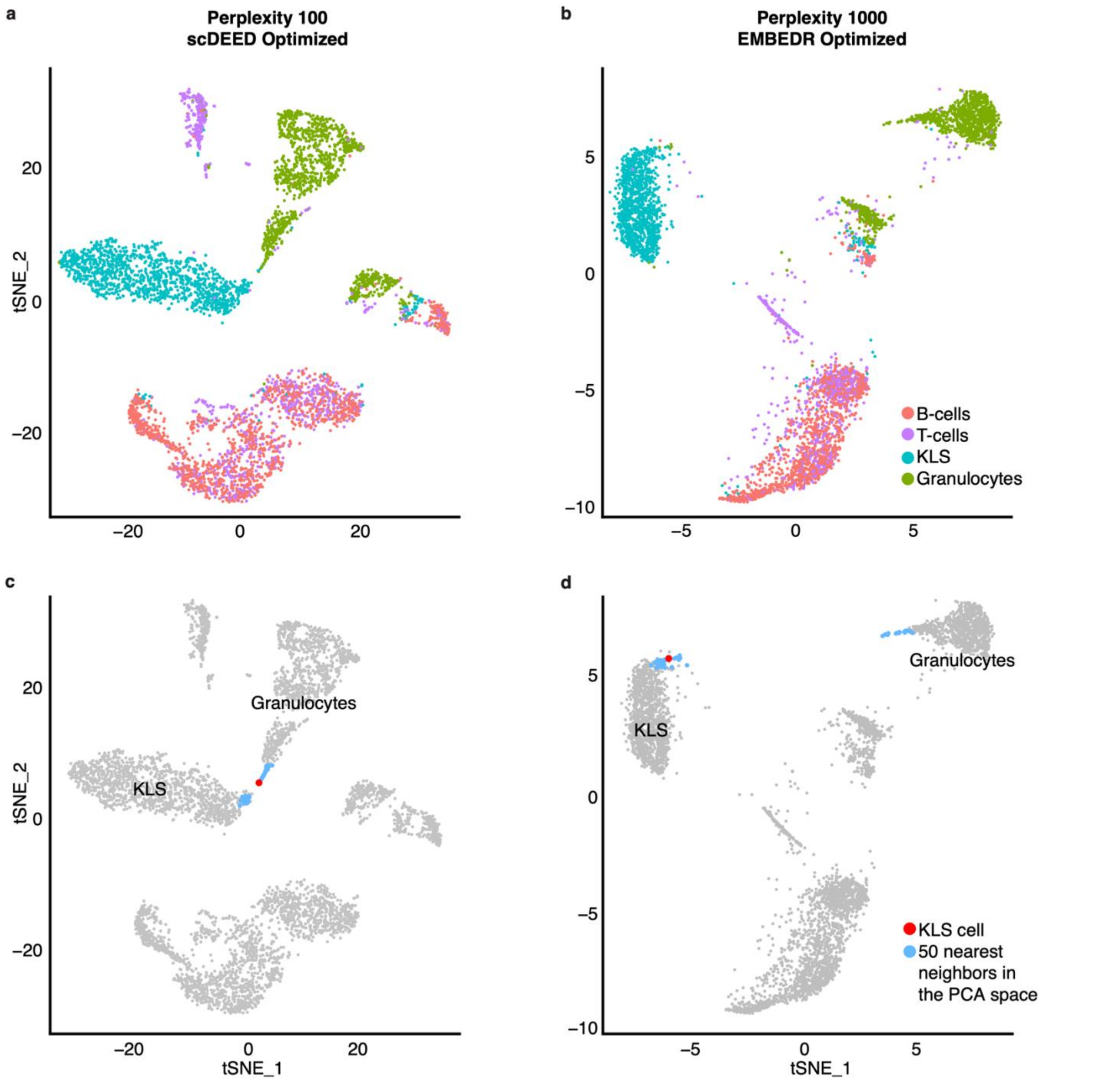

Supplementary Fig. S13. Comparison of scDEED and EMBEDR on the marrow dataset. a–b, t-SNE visualizations at the perplexity of 100 optimized by scDEED (**a**) and the perplexity of 1000 optimized by EMBEDR (**b**). **c–d,** t-SNE visualizations highlighting a random KLS cell's 50 nearest neighbors in the pre-embedding PC space at the optimized perplexity found by scDEED (**c**) or EMBEDR (**d**).

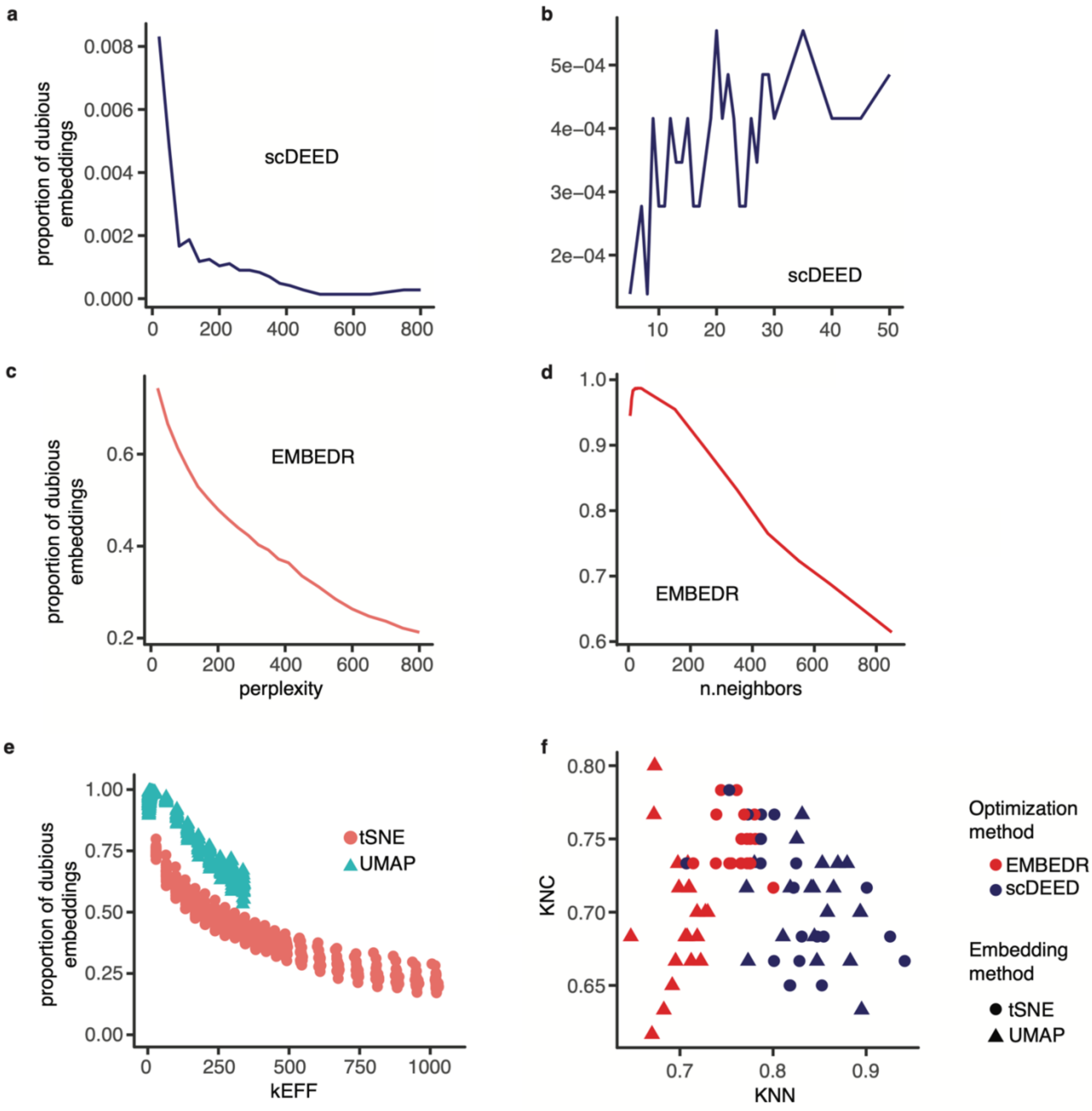

Supplementary Fig. S14. Comparison of scDEED and EMBEDR for optimizing t-SNE and UMAP hyperparameters using simulated data. a–b, Median proportion of dubious embeddings across 20 simulated datasets found by scDEED at varying perplexities for t-SNE (**a**) and n.neighbors for UMAP (**b**). **c–d,** Median proportion of dubious embeddings across 20 simulated datasets found by EMBEDR at varying perplexities for t-SNE (**c**) and n.neighbors for UMAP (**d**). We used exceedingly large n.neighbor values for EMBEDR to show the continual decrease in the number of dubious embeddings. Since scDEED had already achieved 0 dubious embeddings, these larger n.neighbor values were not used for scDEED (**b**). **e,** Proportions of dubious embeddings detected by EMBEDR in t-SNE and UMAP visualizations at each kEff value as calculated in the original paper [4]. Note that UMAP does not have results for large kEFF values because the largest n.neighbors value used in UMAP was 850 (shown in **d**). **f**, Scatterplot of KNC and KNN metrics of the t-SNE and UMAP visualizations (optimized by scDEED and EMBEDR respectively) on the 20 simulated datasets. Source data are provided as Source Data files uploaded on Zenodo.

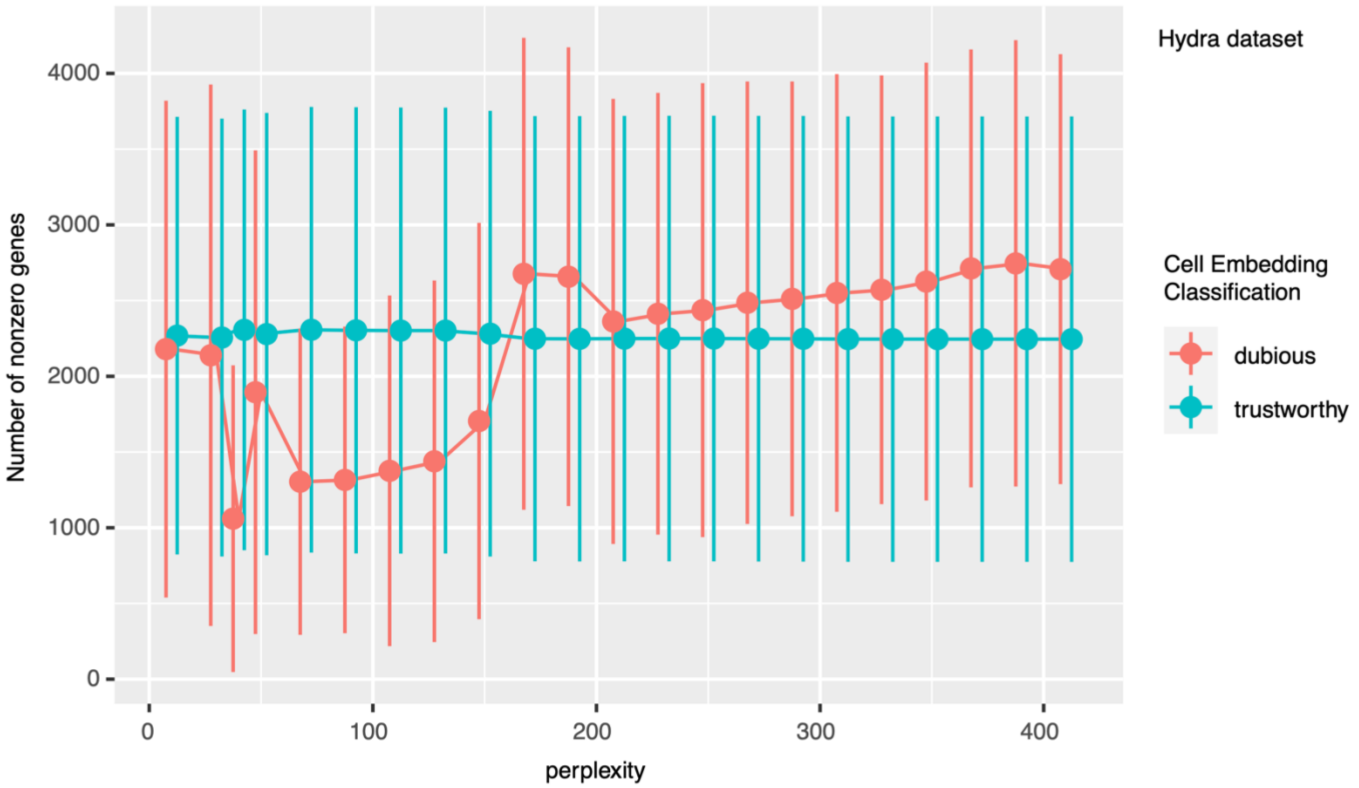

Supplementary Fig. S15: The number of nonzero genes in the cells with dubious or trustworthy embeddings at each of the varying t-SNE perplexity values in the Hydra dataset. Each dot (and its accompanying half-length error bar) indicate the mean (and standard deviation) of the number of nonzero genes in the cells with dubious embeddings or trustworthy embeddings at a given perplexity level. Source data are provided as a Source Data file uploaded on Zenodo.

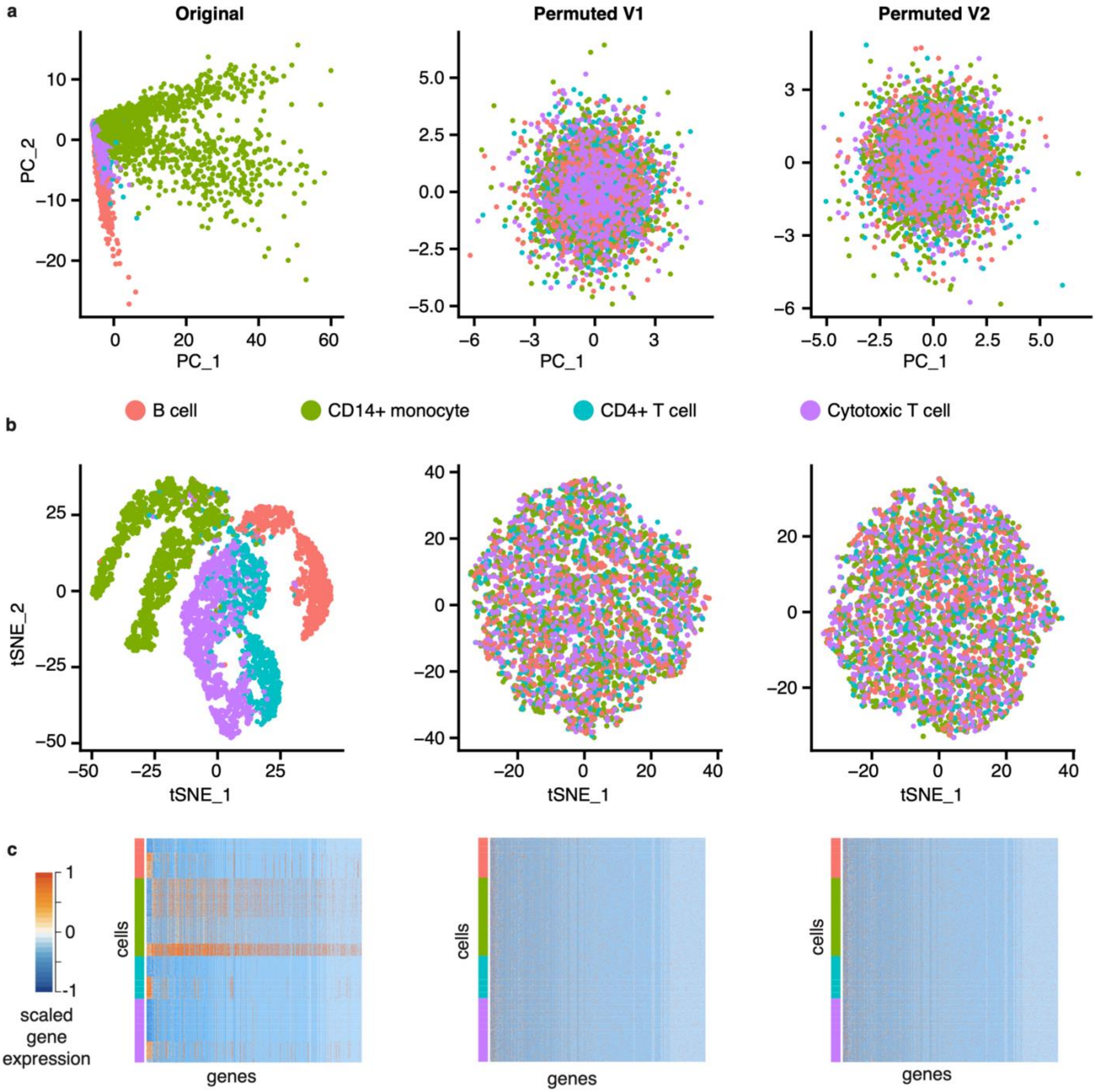

Supplementary Fig. S16. **Permutation removes existing cell-cell relationships in the PBMC inDrops dataset. a**, PCA plots colored by cell type of the inDrops dataset (left), a permuted version of the inDrops dataset (middle), and a second, independently permuted version of the inDrops dataset (right). **b,** t-SNE plots at the original perplexity of 40 for the same three datasets as in **a**. **c,** gene expression heatmaps with the cells grouped by cell type. Note that we truncated scaled expression levels with absolute values above 1 (2% of values) to make a more informative heatmap.

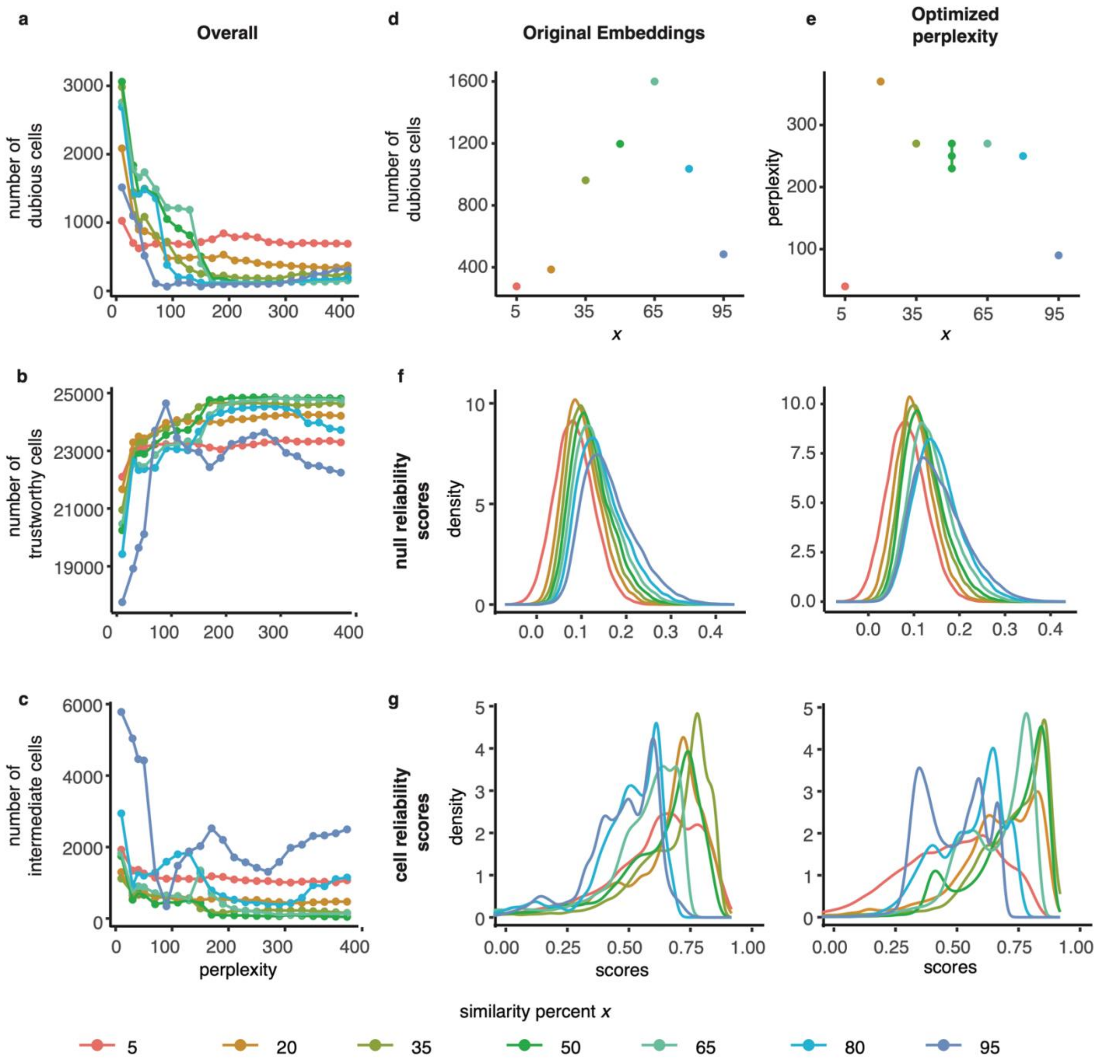

Supplementary Fig. S17. Sensitivity analysis of the "similarity percent" hyperparameter on the Hydra dataset. a–c, Number of dubious (**a**), trustworthy (**b**), or intermediate (**c**) cell embeddings in the Hydra dataset found by scDEED with different "similarity percent" *x* values (corresponding to different colored lines) across t-SNE perplexity values. **d,** Number of dubious embeddings found using the original Hydra embeddings with different *x* values. **e,** Optimized perplexity found by scDEED with different *x* values. **f,** Distribution of the null reliability scores for different *x* found using the original perplexity 40 (left) and the respective optimized perplexity found with *x* (right). **g,** Distribution of the cells' reliability scores for different *x* found using the original embeddings (left) and the embeddings produced by the respective optimized perplexity found with *x* (right). Source data are provided as Source Data files uploaded on Zenodo.

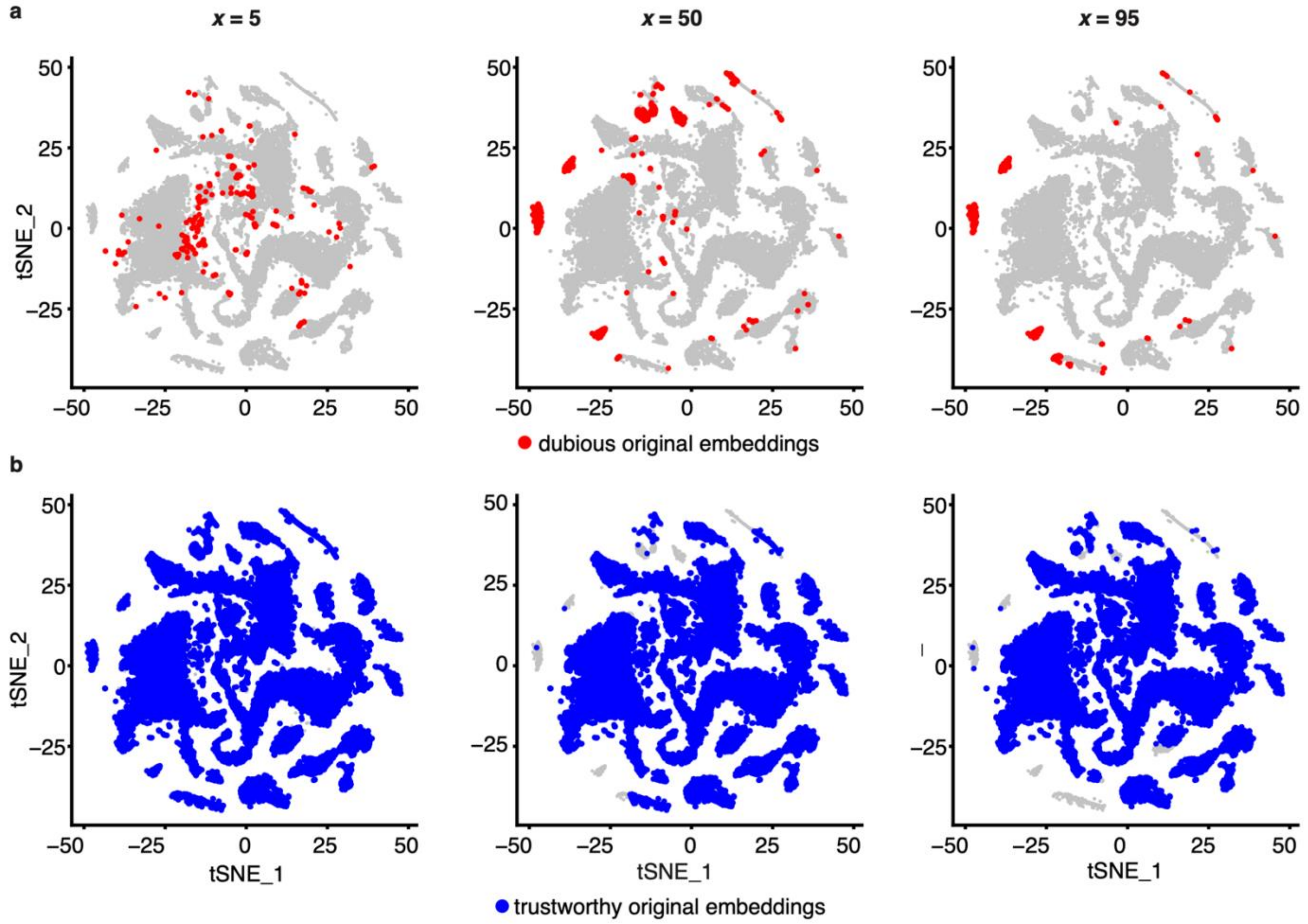

Supplementary Fig. S18. Comparative t-SNE plots of the dubious and trustworthy embeddings detected by scDEED at different similarity percent values on the Hydra dataset. a–b, t-SNE plots of the dubious (**a**) and trustworthy (**b)** cell embeddings found in the original embeddings of the Hydra dataset by scDEED using *x* = 5 (left), 50 (middle), and 95 (right).

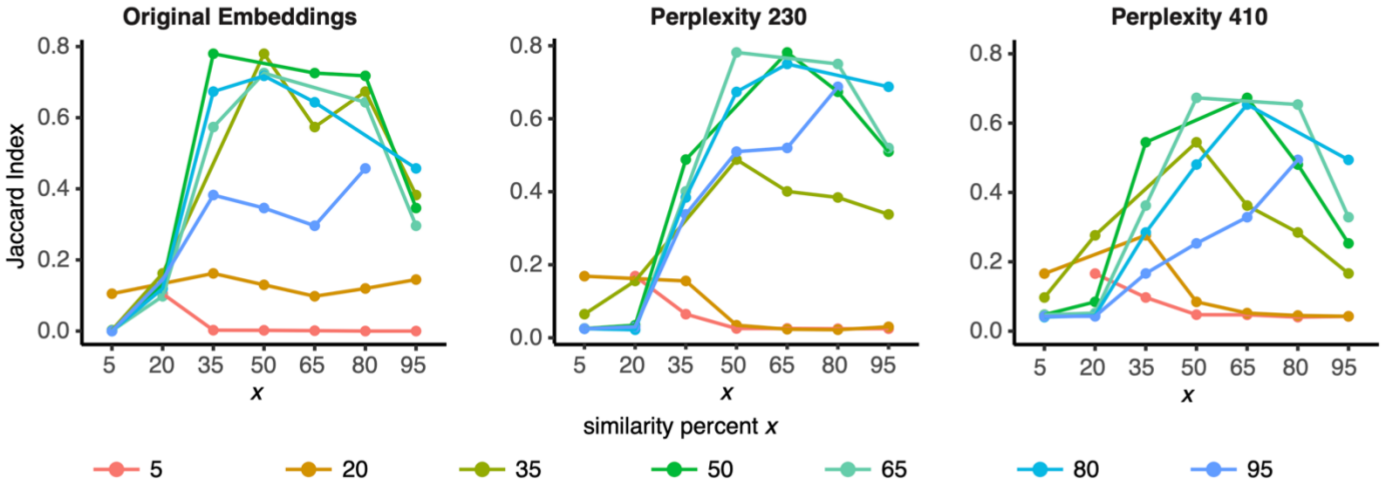

Supplementary Fig. S19. Jaccard indices measuring the similarity of dubious cell embeddings found at different similarity percent values. Jaccard indices measuring the overlaps of dubious cell embeddings found at different *x* (similarity percent) values given a perplexity value of 40 (left; used for the original embeddings), 230 (middle; the scDEED optimized perplexity at *x* = 50) and 410 (right; the maximum candidate perplexity value in the scDEED package). Each horizontal axis value represents one *x* value, and each color/line represents another *x* value. Given a perplexity value, scDEED was applied to detect a set of dubious cell embeddings at each *x* value. A Jaccard index was calculated between every two sets of dubious cell embeddings, which corresponded to two different *x* values (one labeled by the horizontal axis and the other labeled by a color/line). Note that for each horizontal *x* value, we did not calculate the Jaccard index in the line corresponding to the same *x* value because the Jaccard index would be 1; for example, when the horizontal *x* value is 50, no Jaccard index is shown in the green line corresponding to *x* = 50. Source data are provided as a Source Data file uploaded on Zenodo.

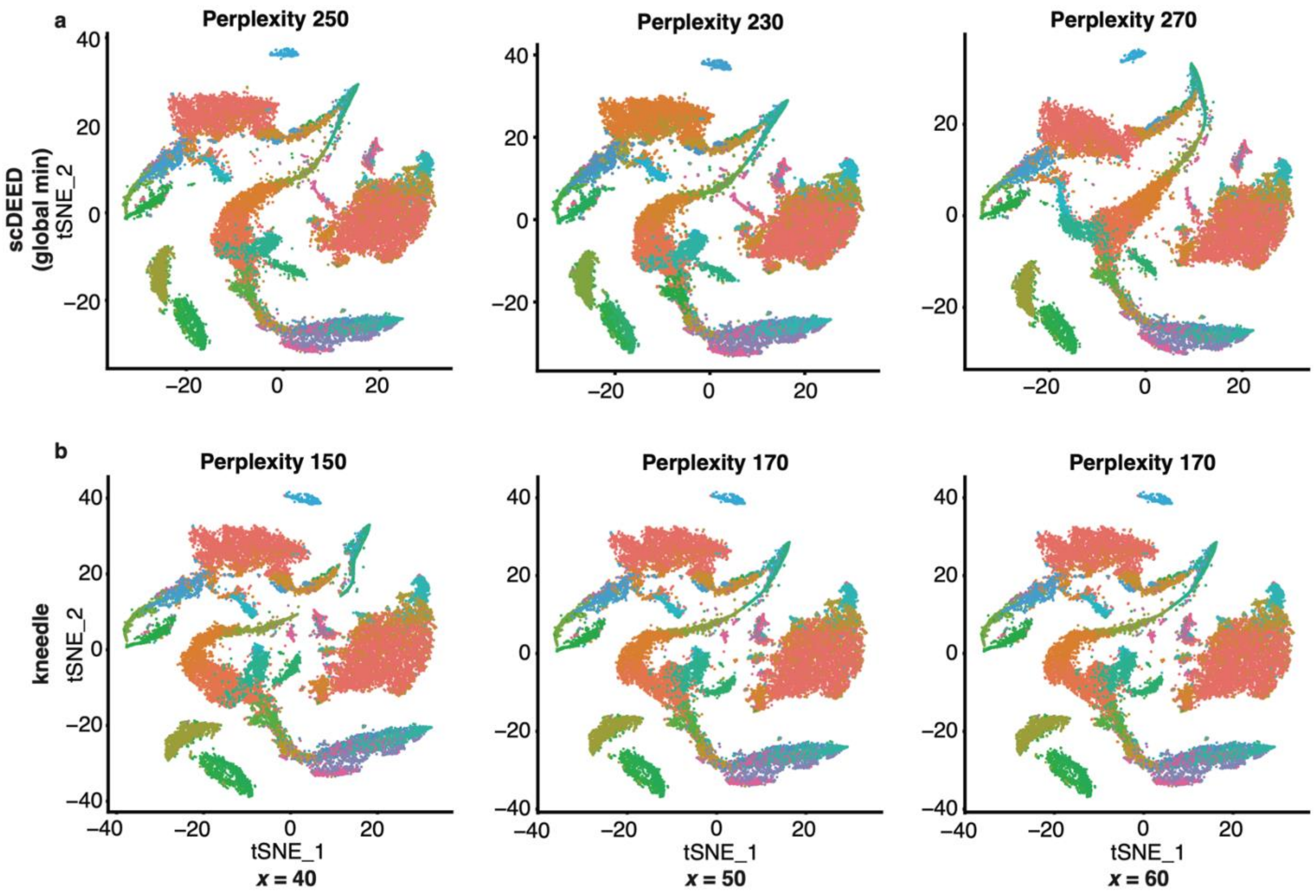

Supplementary Fig. S20: Sensitivity analysis of scDEED in terms of the similarity percent and the optimization criterion. a–b, Comparative t-SNE plots with perplexities optimized by scDEED with the “similarity percent” hyperparameter (*x*, i.e., the percentage of closest neighbors, in Step 4 of the scDEED algorithm) set to *x* = 40, 50, and 60, corresponding to the three columns. Given each *x*, the perplexity was optimized by minimizing the number of dubious cell embeddings (the default approach in scDEED) (**a**) or the “kneedle” method [2] (**b**). For easier visualization, the cell type labels are omitted, and the color key is the same as in Supplementary Fig. S21.

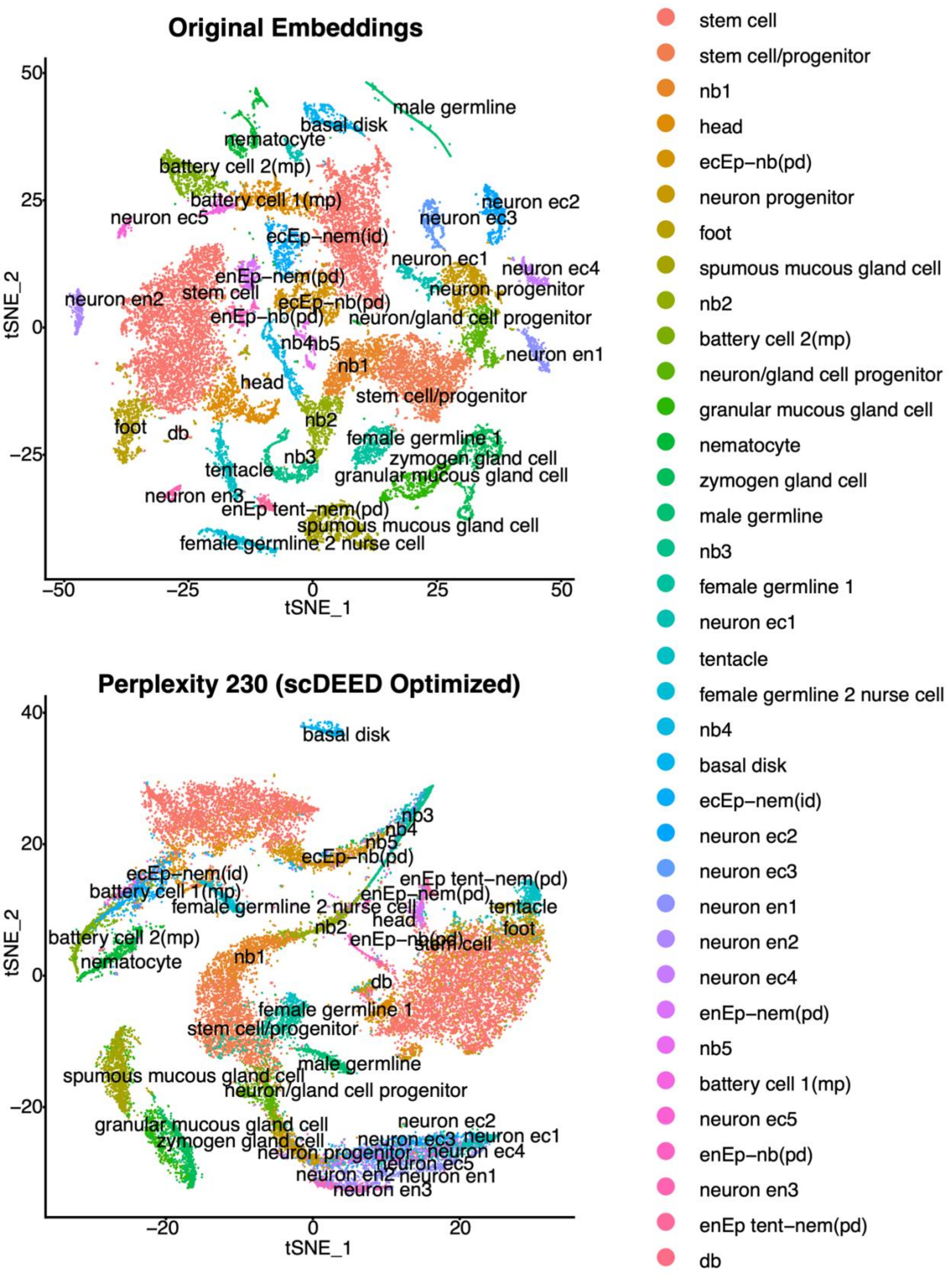

Supplementary Fig. S21. t-SNE plots for the Hydra dataset. Enlarged version of Fig. 2a and 2d with a full legend of cell types.

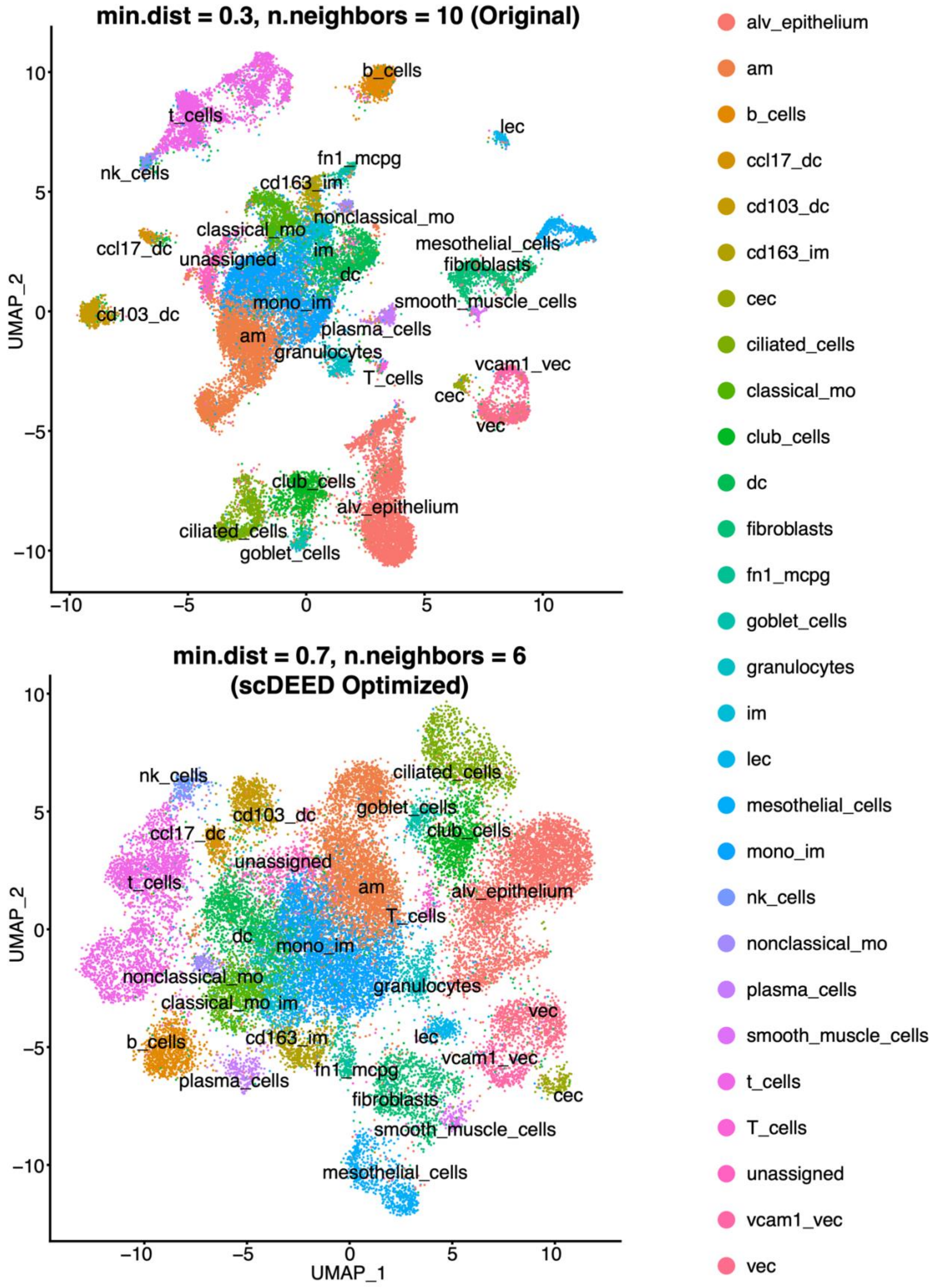

Supplementary Fig S22. UMAP plots for the Alveolar dataset. Enlarged version of Fig. 6a and 6d with a full legend of cell types.

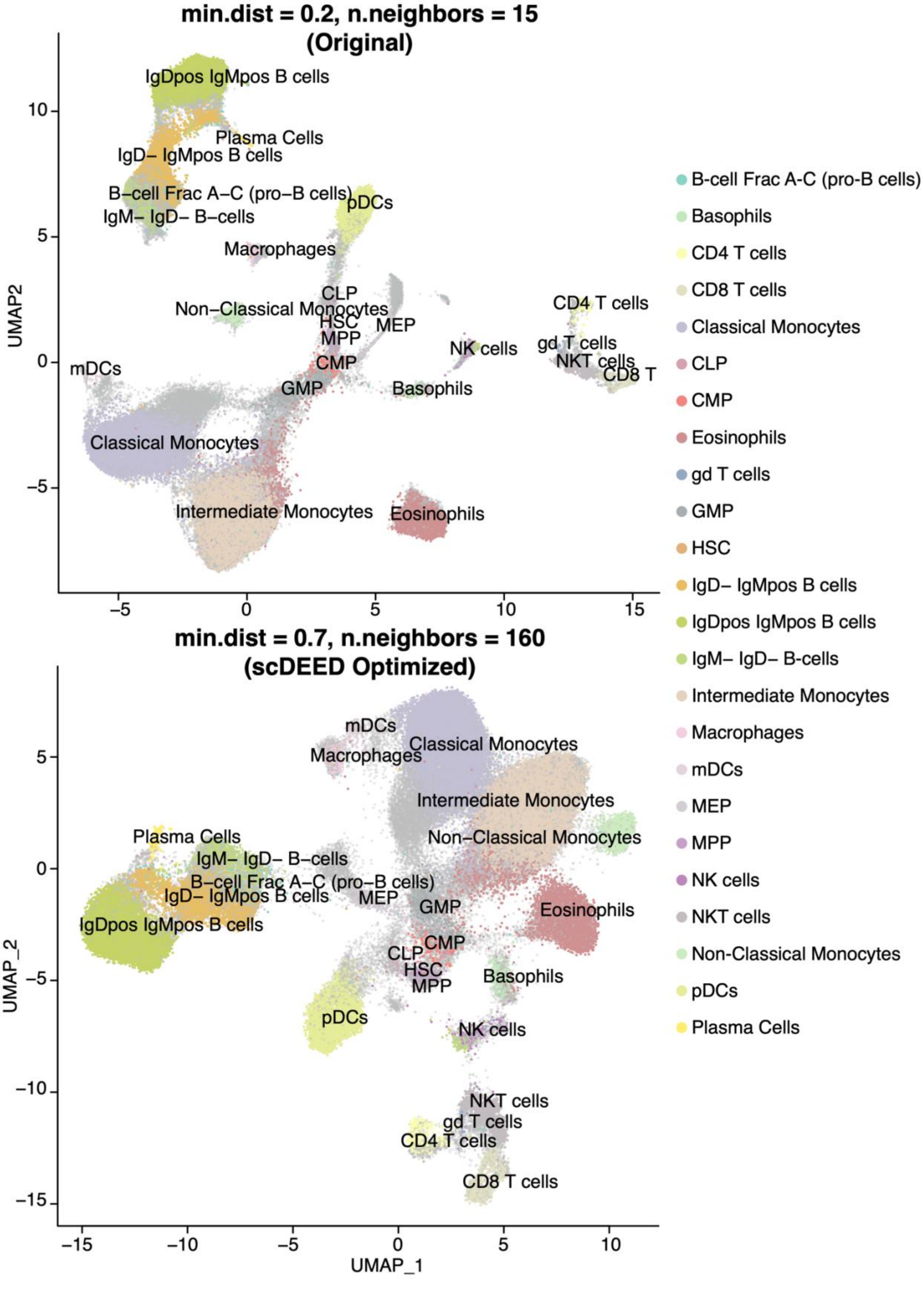

Supplementary Fig. S23. UMAP plots for the Samusik dataset. Enlarged version of Fig. 8a and 8d with a full legend of cell types.

Supplementary Tables

Supplementary Table 1. Differentially expressed (DE) genes between cells with dubious or trustworthy embeddings in cluster 7 of the CAR-T dataset.

Genes highly expressed in cells with dubious embeddings in cluster 7		Genes highly expressed in cells with trustworthy embeddings in cluster 7				
B2M	*IRF2*	*RPL13A*	*RPL21*	*RPS25*	*RPS7*	*NACA*
KLRD1	*IL10RA*	*RPL37*	*RPL36*	*RPS9*	*RPS4X*	*NFKBIA*
DDX5	*SRGN*	*RPS12*	*RPS6*	*RPS3*	*RPS11*	*RPL7*
TXNIP	*RAC2*	*RPL13*	*RPL12*	*RPL14*	*RPS20*	*GLTSCR2*
PRF1	*CFL1*	*RPL19*	*RPS23*	*EEF1D*	*RPS26*	*SNRPD2*
HNRNPA2B1	*IL32*	*RPL11*	*RPL35A*	*RPL38*	*RPL10A*	*RPL36AL*
FTL	*KLRC1*	*RPL32*	*RPS21*	*RPL31*	*RPLP2*	*RPL22*
ARL6IP5	*TTC38*	*RPS15*	*RPS27*	*RPS29*	*RPSA*	*RPL4*
CTSC	*CD53*	*RPL28*	*RPS8*	*RPS16*	*FAU*	*RPL18*
IFITM2	*RABGAP1L*	*RPS18*	*RPL30*	*RPL41*	*RPL27*	*UBA52*
S100A4	*ADIPOR2*	*RPS19*	*RPL18A*	*RPL26*	*RPL8*	*EEF1B2*
C4orf3	*SKAP1*	*RPS3A*	*RPL29*	*RPL24*	*RPS5*	*PFDN5*
S100A11	*TRAF3IP3*	*RPS2*	*RPL35*	*TPT1*	*RPL23*	
RNF213	*IQSEC1*	*RPS15A*	*RPL37A*	*RPL6*	*RPL36A*	
SLC9A3R1	*CLEC2D*	*RPS27A*	*RPL23A*	*RPLP0*	*RPS28*	
IFITM1	*SERINC1*	*RPL34*	*RPS24*	*RPL7A*	*GNB2L1*	

The DE genes were found using `FindMarkers()` function in the R package Seurat with the Wilcoxon rank-sum test (the default setting).

Supplementary References

1. Satopaa V, Albrecht J, Irwin D, Raghavan B. Finding a “Kneedle” in a Haystack: Detecting Knee Points in System Behavior. 2011 31st International Conference on Distributed Computing Systems Workshops. 2011. doi:10.1109/icdcsw.2011.20

2. Chen H, Albergante L, Hsu JY, Lareau CA, Lo Bosco G, Guan J, et al. Single-cell trajectories reconstruction, exploration and mapping of omics data with STREAM. Nat Commun. 2019;10: 1903.

3. La Manno G, Soldatov R, Zeisel A, Braun E, Hochgerner H, Petukhov V, et al. RNA velocity of single cells. Nature. 2018;560: 494–498.

4. Johnson EM, Kath W, Mani M. EMBEDR: Distinguishing signal from noise in single-cell omics data. Patterns (N Y). 2022 Feb 8;3(3):100443. doi: 10.1016/j.patter.2022.100443. PMID: 35510181; PMCID: PMC9058925.
