# Supplementary material for: Statistical method scDEED for detecting dubious 2D single-cell embeddings and optimizing t-SNE and UMAP hyperparameters
Source: Nat Commun. 2024 Feb 26;15:1753. doi: 10.1038/s41467-024-45891-y (PMC10897166; doi:10.1038/s41467-024-45891-y)
Supplement: Supplementary file 3 — Reporting Summary [file 41467_2024_45891_MOESM3_ESM.pdf]

nature portfolio

Corresponding author(s): Jingyi Jessica Li

Last updated by author(s): 9/19/2023

Reporting Summary

Nature Portfolio wishes to improve the reproducibility of the work that we publish. This form provides structure for consistency and transparency in reporting. For further information on Nature Portfolio policies, see our Editorial Policies and the Editorial Policy Checklist.

Statistics

For all statistical analyses, confirm that the following items are present in the figure legend, table legend, main text, or Methods section.

n/a	Confirmed	
☐	☒	The exact sample size (*n*) for each experimental group/condition, given as a discrete number and unit of measurement
☐	☒	A statement on whether measurements were taken from distinct samples or whether the same sample was measured repeatedly
☐	☒	The statistical test(s) used AND whether they are one- or two-sided<br>*Only common tests should be described solely by name; describe more complex techniques in the Methods section.*
☒	☐	A description of all covariates tested
☐	☒	A description of any assumptions or corrections, such as tests of normality and adjustment for multiple comparisons
☐	☒	A full description of the statistical parameters including central tendency (e.g. means) or other basic estimates (e.g. regression coefficient) AND variation (e.g. standard deviation) or associated estimates of uncertainty (e.g. confidence intervals)
☐	☒	For null hypothesis testing, the test statistic (e.g. *F*, *t*, *r*) with confidence intervals, effect sizes, degrees of freedom and *P* value noted<br>*Give P values as exact values whenever suitable.*
☒	☐	For Bayesian analysis, information on the choice of priors and Markov chain Monte Carlo settings
☐	☒	For hierarchical and complex designs, identification of the appropriate level for tests and full reporting of outcomes
☐	☒	Estimates of effect sizes (e.g. Cohen's *d*, Pearson's *r*), indicating how they were calculated

Our web collection on statistics for biologists contains articles on many of the points above.

Software and code

Policy information about availability of computer code

Data collection	Hydra dataset: obtained under accession code GSE121617, https://www.ncbi.nlm.nih.gov/geo/query/acc.cgi?acc=GSE121617.<br>CAR-T dataset: obtained under accession code GSE125881, https://www.ncbi.nlm.nih.gov/geo/query/acc.cgi?acc=GSE125881.<br>Alveolar dataset: obtained under accession code GSE141259, https://www.ncbi.nlm.nih.gov/geo/query/acc.cgi?acc=GSE141259.<br>Samusik dataset: obtained from https://figshare.com/s/9c3a0136f12b97f1dadd<br>Human PBMC dataset: obtained under accession number GSE132044, through the SeuratData package, version 0.2.2<br>Marrow dataset: obtained from Tabular Muris, from link: https://figshare.com/projects/Tabula_Muris_Transcriptomic_characterization_of_20_organs_and_tissues_from_Mus_musculus_at_single_cell_resolution/27733<br>DG dataset: obtained from http://pklab.med.harvard.edu/velocyto/DG1/10X43_1.loom<br>Simulated Data: trained from data obtained from GSE92332, https://www.ncbi.nlm.nih.gov/geo/query/acc.cgi?acc=GSE92332.<br>The computer code and processed data are available at Zenodo https://zenodo.org/record/7216361#.ZDNgd-zMLJ8<br><br>R Core Team (2021). R: A language and environment for statistical computing. R Foundation for Statistical Computing, Vienna, Austria. URL https://www.R-project.org/.

Data analysis	Seurat version 3.2.3, SeuratData version 0.2.2, doParallel version 1.0.15, foreach version 1.5.0, ggsci version 2.9, Rogue version 2.0.0, distances version 0.1.8, velocyto.R version 0.6, Rfast version 1.9.9; VGAM version 1.1.3; pracma version 2.2.9; ggplot2 version 3.3.2; SeuratWrappers version 0.3.0, scDesign3 version 0.99.6<br>R Core Team (2021). R: A language and environment for statistical computing. R Foundation for Statistical Computing, Vienna, Austria. URL https://www.R-project.org/.

For manuscripts utilizing custom algorithms or software that are central to the research but not yet described in published literature, software must be made available to editors and reviewers. We strongly encourage code deposition in a community repository (e.g. GitHub). See the Nature Portfolio guidelines for submitting code & software for further information.

Data

Policy information about availability of data

All manuscripts must include a data availability statement. This statement should provide the following information, where applicable:

- Accession codes, unique identifiers, or web links for publicly available datasets
- A description of any restrictions on data availability
- For clinical datasets or third party data, please ensure that the statement adheres to our policy

Hydra dataset: obtained under accession code GSE121617, https://www.ncbi.nlm.nih.gov/geo/query/acc.cgi?acc=GSE121617.
CAR-T dataset: obtained under accession code GSE125881, https://www.ncbi.nlm.nih.gov/geo/query/acc.cgi?acc=GSE125881.
Alveolar dataset: obtained under accession code GSE141259, https://www.ncbi.nlm.nih.gov/geo/query/acc.cgi?acc=GSE141259.
Samusik dataset: obtained from https://figshare.com/s/9c3a0136f12b97f1dadd
Human PBMC dataset: obtained under accession number GSE132044, through the SeuratData package, version 0.2.2
Marrow dataset: obtained from Tabular Muris, from link: https://figshare.com/projects/Tabula_Muris_Transcriptomic_characterization_of_20_organs_and_tissues_from_Mus_musculus_at_single_cell_resolution/27733
DG dataset: obtained from http://pklab.med.harvard.edu/velocyto/DG1/10X43_1.loom
Simulated Data: trained from data obtained from GSE92332, https://www.ncbi.nlm.nih.gov/geo/query/acc.cgi?acc=GSE92332.
The computer code and processed data are available at Zenodo https://zenodo.org/record/7216361#.ZDNgd-zMLJ8

Research involving human participants, their data, or biological material

Policy information about studies with human participants or human data. See also policy information about sex, gender (identity/presentation), and sexual orientation and race, ethnicity and racism.

Reporting on sex and gender	N/A
Reporting on race, ethnicity, or other socially relevant groupings	N/A
Population characteristics	N/A
Recruitment	N/A
Ethics oversight	N/A

Note that full information on the approval of the study protocol must also be provided in the manuscript.

Field-specific reporting

Please select the one below that is the best fit for your research. If you are not sure, read the appropriate sections before making your selection.

☒ Life sciences ☐ Behavioural & social sciences ☐ Ecological, evolutionary & environmental sciences

For a reference copy of the document with all sections, see nature.com/documents/nr-reporting-summary-flat.pdf

Life sciences study design

All studies must disclose on these points even when the disclosure is negative.

Sample size	Sample size was not calculated beforehand. We used publicly available datasets. Cell count was determined by experimental design, often limited by the number of samples that could be gathered or sequencing cost restrictions. Since this is a methods paper to optimize visualization methods, the cell count should be representative of real data set sizes. We have many datasets, including small sized datasets like the dentate gyrus dataset with n = 3,396 cells, and large datasets like Samusk_01 with n = 841,644 cells.
Data exclusions	Data was processed according to original papers where they were published. In general, cells are excluded for low gene count or high proportions of mitochondrial genes.
Replication	We replicated the success of scDEED by verifying its success on multiple datasets. Each was performed independently.

Randomization	We did not have experimental groups; randomization was not done.
Blinding	Blinding not relevant; no experimental groups.

Reporting for specific materials, systems and methods

We require information from authors about some types of materials, experimental systems and methods used in many studies. Here, indicate whether each material, system or method listed is relevant to your study. If you are not sure if a list item applies to your research, read the appropriate section before selecting a response.

Materials & experimental systems

n/a	Involved in the study	
☒	☐	Antibodies
☒	☐	Eukaryotic cell lines
☒	☐	Palaeontology and archaeology
☒	☐	Animals and other organisms
☒	☐	Clinical data
☒	☐	Dual use research of concern
☒	☐	Plants

Methods

n/a	Involved in the study	
☒	☐	ChIP-seq
☒	☐	Flow cytometry
☒	☐	MRI-based neuroimaging
